# Paternal genetic diversity, differentiation and phylogeny of three white yak breeds/populations in China

**DOI:** 10.1038/s41598-022-23453-w

**Published:** 2022-11-11

**Authors:** Jing Luo, Xudong Wei, Wenxian Liu, Shengmei Chen, Zulfiqar Ahmed, Wenping Sun, Chuzhao Lei, Zhijie Ma

**Affiliations:** 1 Academy of Animal Science and Veterinary Medicine, Qinghai University, No. 1 Weier Road, Bio-Science Industrial District, Xining, 810016 Qinghai China; 2 Key Laboratory of Animal Genetics and Breeding on Tibetan Plateau, Ministry of Agriculture and Rural Affairs, Xining, 810016 China; 3 Plateau Livestock Genetic Resources Protection and Innovative Utilization Key Laboratory of Qinghai Province, Xining, 810016 China; 4 Animal Disease Prevention and Control Center of Menyuan County in Qinghai Province, Menyuan, 810399 Qinghai China; 5 Faculty of Veterinary and Animal Sciences, University of Poonch Rawalakot, Azad Jammu & Kashmir, 12350 Pakistan; 6 Bureau of Agriculture, Animal Husbandry and Rural Science and Technology of Jiulong County in Sichuan Province, Jiulong, 626199 Sichuan China; 7 College of Animal Science and Technology, Northwest A&F University, Xianyang, 712100 China

**Keywords:** Genetics, Molecular biology, Zoology

## Abstract

The white yak, a type of unique and valuable farm animals on the Qinghai-Tibet Plateau, are mainly distributed in Tianzhu (County of Gansu Province), Menyuan, Huzhu and Ledu (three Counties of Qinghai Province) in China. In the present study, the Y-chromosomal genetic diversity, differentiation and phylogeny of three Chinese white yak breeds/populations (Tianzhu, Huzhu and Menyuan) were comprehensively explored using five Y-SNPs (*SRY4*, *USP9Y*, *UTY19*, *AMELY3* and *OFD1Y10*) and one Y-STR (*INRA189*) markers. The results showed that six Y-haplotypes (H1Y1, H9Y1, H10Y1, H11Y2, H12Y2 and H13Y2) were identified in 97 male yak from three white yak breeds/populations. Among these haplotypes, H1Y1, H10Y1 and H11Y2 were shared by all of breeds/populations and H12Y2 was shared by Tianzhu and Huzhu populations. However, H9Y1 and H13Y2 haplotypes were only detected in Menyuan and Tianzhu white yak populations, respectively. The Y-haplotype diversity was maximum in Huzhu white yak (0.7500 ± 0.0349), the medium in Tianzhu white yak (0.6881 ± 0.0614) and the lowest in Menyuan white yak (0.5720 ± 0.0657). The total Y-haplotype diversity of three white yak breeds/populations was 0.7567 ± 0.0233, indicating rich paternal genetic diversity in white yak. The *F*_*ST*_ values showed a moderate differentiation between Tianzhu and Menyuan (*F*_*ST*_ = 0.0763, P < 0.05) populations, but a weak differentiation between Huzhu and Tianzhu white yak breeds/populations (*F*_*ST*_ = 0.0186, P > 0.05) and Huzhu and Menyuan (*F*_*ST*_ = − 0.005, P > 0.05) populations. The clustering analysis revealed a close genetic relationship between Huzhu and Menyuan white yak, both were far from Tianzhu white yak breed. The phylogenetic analyses showed that white yak had two Y-haplogroups/lineages (Y1 and Y2) with two potential paternal origins. The findings of present study provide new insight into the basic information for the formulation of molecular breeding programs of white yak. Moreover, it also contributes to the conservation and utilization of this special animal genetic resource.

## Introduction

Yak (*Bos grunniens*) is a unique bovine specie that inhabits on the Qinghai-Tibet plateau (QTP) and adjacent mountains or subalpine mountainous regions in China. It is known for its better adaptability to the harsh environmental conditions such as high altitude, severe cold, strong ultraviolet radiations, scarce forage and deprived oxygen level^1^. The white yak, a rare ecological type in domestic yak, produces two types of white fiber (coarse outer hair and a fine down fiber) with relatively high economic value^2^. Since the 1970s, the down fiber has been used extensively by the textile industry as an alternative to other fine animal fibers in China^1^. Currently, the white yak is mainly distributed on both sides of the Qilian Mountains on QTP, including Tianzhu County in Gansu Province, and Huzhu, Menyuan and Ledu Counties in Qinghai Province of China^3^.

The mammalian Y chromosome is considered as a symbol of maleness with the characteristics of paternal inheritance, lower mutation rate, and less susceptibility to recombination and reverse mutation^4,5^. It has been used to explore the paternal genetic diversity, origination and population genetic structure of domestic animals. In recent years, two kinds of molecular markers, including Y chromosome microsatellites (Y-STRs) and single nucleotide polymorphisms (Y-SNPs), have been used to investigate the yak paternal population genetics, which showed that Chinese yak breeds/populations owned rich paternal genetic diversity with two different haplogroups/lineages^6–10^. In our recent maternal genetic study on white yak, we found that each of the three Chinese white yak breeds/populations (Menyuan, Huzhu and Tianzhu) carried special maternal genetic information with relatively rich genetic diversity^11^. However, to date, there is no comprehensive paternal genetic analysis of these three Chinese white yak breeds/populations in literature. In this context, the present study explored the Y chromosomal genetic diversity, differentiation, and phylogeny of three Chinese white yak breeds/populations. The Y-SNPs and Y-STR markers were used to clarify the status of white yak genetic resources and to provide theoretical basis for the protection and utilization of white yak populations for future breeding plans and policies.

## Materials and methods

### Ethical approval

Based on the recommendations of the Regulations for the Administration of Affairs Concerning Experimental Animals of China, the Institutional Animal Care and Use Committee of the Academy of Animal Science and Veterinary Medicine, Qinghai University approved all the animal experiments used in this study. Specific consent procedures were not required for this study following the recommendation of the Regulations for the Administration of Affairs Concerning Experimental Animals of China.

### Sample collection and genomic DNA extraction

**Figure 1:**
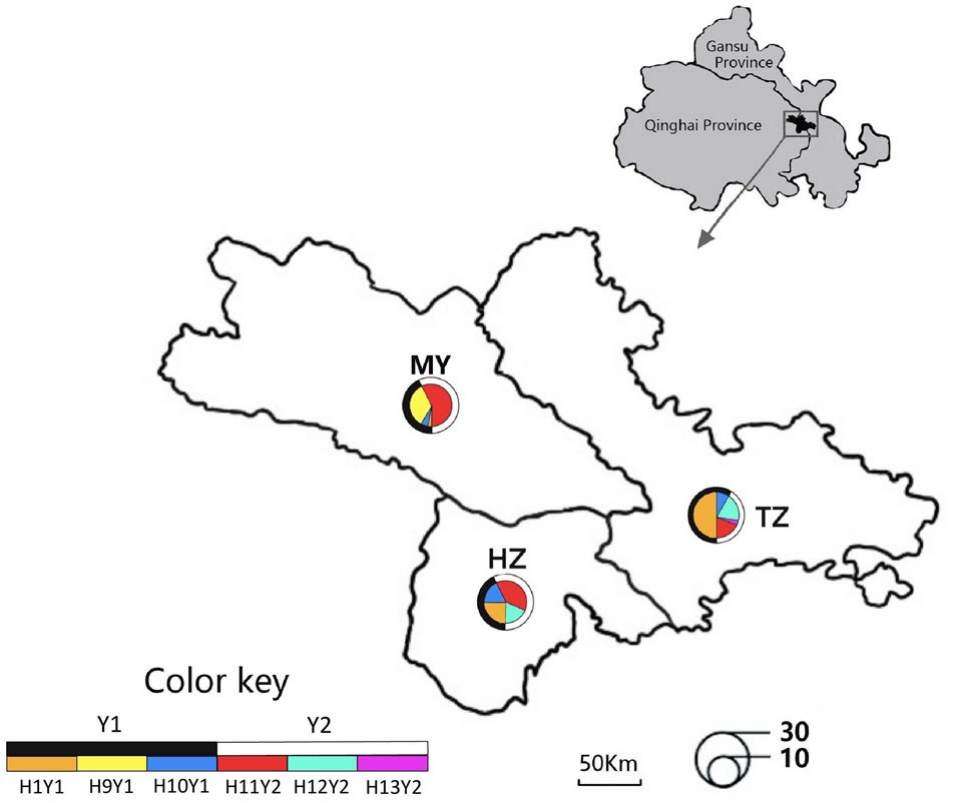
Sampling sites and haplotype/haplogroup distributions of three Chinese white yak breeds/populations. Maps is created using ArcGIS software. Note: TZ, Tianzhu white yak; MY, Menyuan white yak; HZ, Huzhu white yak. The shaded grey area shows Gansu Province and Qinghai Province of China, and the shaded black area within grey area represents Tianzhu County in Gansu Province, and Huzhu and Menyuan Counties in Qinghai Province. The different colors in each circle represent different haplotypes/haplogroups identified. The size of the circle is proportional to the number of individuals. The distances among breeds/populations were measured from the center of the pie charts.

The blood samples from 34, 32, and 31 male white yak were randomly collected by jugular venipuncture from the core breeding tracts of Tianzhu County of Gansu Province, Huzhu and Menyuan Counties of Qinghai Province in China, respectively (Table 1, Fig. 1). The genomic DNA was extracted using blood DNA extraction kit (Aidlab Biotechnologies Co., Ltd, China) and stored at – 20 ℃ for future use.

**Table 1:** Frequencies and diversities of haplotypes in three Chinese white yak breeds/populations.

Breeds/Populations	No. of samples	Haplotypes/haplogroups						Nh	Hd ± SD
		Y1			Y2				
		H1Y1	H9Y1	H10Y1	H11Y2	H12Y2	H13Y2		
Tianzhu	34	0.500 (17)		0.088 (3)	0.177 (6)	0.206 (7)	0.029 (1)	5	0.6881 ± 0.0614
Huzhu	32	0.250 (8)		0.188 (6)	0.374(12)	0.188(6)		4	0.7500 ± 0.0349
Menyuan	31	0.032 (1)	0.323 (10)	0.064 (2)	0.581(18)			4	0.5720 ± 0.0657
Total	97	0.268 (26)	0.103 (10)	0.114 (11)	0.371(36)	0.134(13)	0.010(1)	6	0.7567 ± 0.0233

Nh, number of haplotypes; Hd, haplotype diversity; SD, standard deviation.

### PCR amplification, sequencing and data analysis

By referring to the reported information on five Y-SNPs (*SRY4*, *USP9Y*, *UTY19*, *AMELY3*, and *OFD1Y10*) and one Y-STR (*INRA189*)^7,9,10,12^, the six pairs of primers were synthesized and then used to amplify these markers as previously stated. The purified PCR products were sequenced and typed commercially (Shanghai Sangon Biotech Co., Ltd). The CHROMAS 2.6.6 (Technelysium Pty Ltd, South Brisbane, Australia) was used for sequencing output, while, BIOEDIT 7.2.5 was used for multiple sequence alignment^13,14^. Meanwhile, the typing results of Y-STR *INRA189* were analyzed with GENEMARKER 1.91 to determine its allele size^15^. The Y chromosome haplotype/haplogroup (Y-haplotype/haplogroup) of each male white yak was determined by combined analysis of the above two kinds of molecular markers. The number of Y chromosome haplotypes of white yak breeds/populations was determined by DNASP 5.10.01 and ARLEQUIN 3.11 software^16,17^. Sampling sites and haplotype distributions maps were created using ArcGIS 10.7 to show geographic relationship. In addition, the Y chromosome haplotype diversity (Hd ± SD) was calculated to evaluate the paternal genetic diversity of three white yak breeds/populations. The pairwise fixation index (*F*_*ST*_) values were calculated using ARLEQUIN 3.11 software to indicate the differentiation degree amongst the breeds/populations. The UPGMA (unweighted pair-group method with arithmetic means, UPGMA) tree was constructed using MEGA 7.0 based on *F*_*ST*_ to reveal genetic relationships among white yak breeds/populations^18^. Based on nucleotide variations between different haplotypes, the median-joining (MJ) network was drawn using NETWORK 10.1 to reveal the phylogenetic relationship among haplotypes/haplogroups^19^.

## Results and discussion

### Identification of Y-haplotype/haplogroup in white yak

The amplified fragment lengths of the five Y-SNPs (*SRY4*, *USP9Y*, *UTY19*, *AMELY3*, and *OFD1Y10*) were 969 bp, 470 bp, 290 bp, 971 bp and 763 bp, respectively. The corresponding sequences had been submitted to GenBank (Accession No. *SRY4*: MF683848, *USP9Y*: MF683849, *UTY19*: MF683850, *AMELY3*: MF683851, and *OFD1Y10*: MF683852). The sequencing results of each of the Y-SNP were analyzed by multiple sequence alignment and 10 previously reported Y-SNPs were also detected (Table S1)^9,10^. In the typing analysis of the Y-STR *INRA189* marker, three alleles of 155 bp, 157 bp and 161 bp were detected. Notably, the allele of 161 bp was only detected in Tianzhu white yak, while, others were found in all three breeds/populations (Table S1).

The haplotypes/haplogroups were jointly identified based on the alleles of Y-SNPs and Y-STR. According to the previous judgment criterion for yak Y-haplotype^10^, a total of six Y-haplotypes were determined in three white yak breeds/populations, namely H1Y1, H9Y1, H10Y1, H11Y2, H12Y2, and H13Y2 (Table 1, Table S1). The six Y-haplotypes could be further divided into two Y-haplogroups (Y1 and Y2): Y1 haplogroup included three haplotypes (H1Y1, H9Y1, and H10Y1), whereas, the Y2 haplogroup contained H11Y2, H12Y2 and H13Y2 haplotypes (Table S1).

### Y-chromosome haplotype diversity of white yak

The frequencies of six Y-haplotypes were different among three white yak breeds/populations (Fig. 1, Table 1). In total, the H11Y2 haplotype was predominant (37.1%) and H13Y2 (1.0%) was scarce in the white yak. Three haplotypes (H1Y1, H10Y1, and H11Y2) were common and shared by all three white yak breeds/populations; however, H12Y2 was just shared by Tianzhu and Huzhu white yak populations. The H9Y1 and H13Y2 haplotypes were exclusively found in Menyuan and Tianzhu white yak, respectively. These results showed that Menyuan and Tianzhu white yak breeds/populations possessed unique paternal genetic information. In our previous study, 5, 3, and 43 specific maternal haplotypes were detected in Menyuan, Huzhu and Tianzhu white yak breeds/populations, respectively^11^. Based on the studies of maternal and paternal genetic markers, it can be concluded that Menyuan and Tianzhu white yak populations possessed specific genetic information; therefore, the conservation and application should be independently carried for these genetic units in the future.

In this study, the total haplotype diversity of three white yak breeds/populations was 0.7567 ± 0.0233. Comparing to the previous report^10^, it showed that the white yak had higher total haplotype diversity (0.7567 ± 0.0233) than 15 other Chinese domestic yak breeds/populations (0.6946 ± 0.0143), but lower than the wild yak population (0.8214 ± 0.1007). This total haplotype diversity revealed that white yak also owned rich paternal genetic diversity. The haplotype diversities of Huzhu, Tianzhu, and Menyuan white yak breeds/populations in this study were 0.7500 ± 0.0349, 0.6881 ± 0.0614 and 0.5720 ± 0.0657, respectively (Table 1). It indicated that the Y-haplotype diversity was maximum in Huzhu white yak but the lowest in Menyuan white yak. Surprisingly, the haplotype diversity of the Huzhu white yak was higher than that of the other previously reported Chinese domestic yak breeds/populations (0.1174–0.7273) but only lower than that of the wild yak population (0.8214 ± 0.1007)^10^. At the same time, the haplotype diversities of Menyuan and Tianzhu white yak breeds/populations were also higher than that of the most Chinese domestic yak breeds/populations (0.1174–0.7273)^10^. The present results indicated a rich paternal genetic diversity in all three Chinese white yak breeds/populations. While, Huzhu white yak showed the highest level of paternal genetic diversity.

### Differentiation and clustering relationship among white yak populations

The genetic differentiation index (*F*_*ST*_) was used to evaluate the differentiation degree among white yak breeds/populations. The *F*_*ST*_ values ranged from − 0.0050 to  0.0763, whereas, *R*_*ST*_ values (linearized *F*_*ST*_ values) ranged from 0 to 0.0826 (Table S2), indicating the variable differentiation degree amongst the three white yak breeds/populations. Refer to Wright's standard^20^, the *F*_*ST*_ values indicated moderate differentiation between Tianzhu and Menyuan white yak breeds/populations (0.0763, P < 0.05), however, the Huzhu white yak population displayed a weaker genetic differentiation from Tianzhu white yak (0.0186, P > 0.05) and Menyuan white yak (-0.0050, P > 0.05) (Table S2). The differentiation among three white yak breeds/populations might have been resulted from the differences in the diversified living environment, geographic isolation and human selection.

The UPGMA tree was constructed using *R*_*ST*_ values among populations for cluster analysis (Fig. S1). The result showed that Huzhu and Menyuan white yak populations clustered together first and then with Tianzhu white yak. The results of the clustering relationship revealed that there was a close genetic relationship between Huzhu and Menyuan white yak but far genetic relationship with Tianzhu white yak.

### Phylogenetic network analysis of white yak

**Figure 2:**
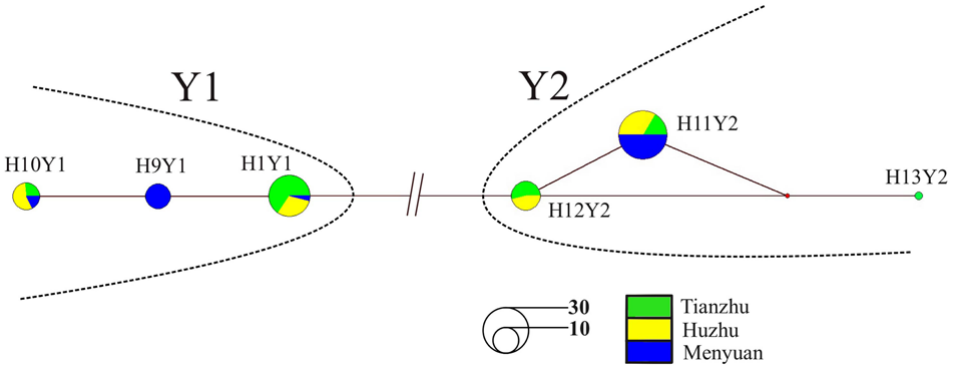
Median-joining network of six Y-haplotypes identified in three Chinese white yak breeds/populations based on Y-SNP and Y-STR makers. Colors correspond to different breeds/populations. The size of each haplotype circle denotes the number of observed individuals.

The network analysis showed that six Y-haplotypes were divided into two haplogroups/lineages (Y1 and Y2), suggesting two paternal origins of the white yak. The current observation is consistent with the previous research findings on wild and other domestic yak breeds^10^. In the present study, Y1 and Y2 lineages had three haplotypes in each lineage (Fig. 2). The Y1 haplogroup/lineage was observed in 48.45% (47/97) individuals and the Y2 haplogroup/lineage in 51.55% (50/97) individuals (Table 1), indicating that Y2 was a dominant haplogroup/lineage in white yak. Meanwhile, the proportions of Y1 in Menyuan, Huzhu, and Tianzhu white yak were 41.94%, 43.75%, and 58.82%, respectively; however, the proportions of Y2 in Menyuan, Huzhu, and Tianzhu white yak were observed to be 58.06%, 56.25%, and 41.18%, respectively (Table 1). The observed data indicated the dominance of the Y1 haplogroup/lineage in the Tianzhu white yak and Y2 haplogroup/lineage in the Menyuan and Huzhu white yak populations. Previous studies represented that the Y1 haplogroup/lineage was dominant haplogroup/lineage in most of the domestic and wild yak populations except for the Pali yak breed^10^. Therefore, our present findings showed that the population structure composition of Menyuan and Huzhu white yak is different from most of the other Chinese domestic yak breeds but similar to the Pali yak breed. Further exploration at the genome level would be needed to unravel the genomic differences among yak breeds/populations.

## Conclusion

In conclusion, the white yak in China showed a rich paternal genetic diversity. The Menyuan and Tianzhu white yak breeds/populations possessed unique haplotypes with a medium differentiation level. The Huzhu white yak and Menyuan white yak had a closer genetic relationship, but they both had far relationships to Tianzhu white yak. The Chinese white yak owned two haplogroups/lineages of Y1 and Y2, an indication of two paternal origins. The population structure composition of Menyuan and Huzhu white yak was different from most of other Chinese domestic yak breeds but similar to Pali yak breed. Given paternal genetic diversity, genetic differentiation and clustering relationship among white yak populations, it is suggested that the conservation of white yak genetic resources should be strengthened to protect their unique and excellent genetic characteristics and to make reasonable utilization. The Huzhu and Menyuan white yak in Qinghai Province and Tianzhu white yak in Gansu Province should be considered as different genetic units.

## Supplementary Information

Supplementary Figure S1.

Supplementary Table S1.

Supplementary Table S2.

## Data Availability

The executable codes and datasets are available from the corresponding author on reasonable request.

## References

[1] Wiener, G., Han, J. L., Long, R. J. The Yak. In *Bangkok: The Regional Office for Asia and the Pacific of the Food and Agriculture Organization of the United Nations* (2003).

[2] Yan P, Xiaoyun W, Yaqin G (2019). Analysis on the development status of yak plush industry. China Econ. Trade Herald..

[3] Compilation Committee of animal and poultry records and maps of Qinghai Province. In *Animal and Poultry Breeds of Qinghai Province, Qinghai People’s Publishing House* 52–54 (1983) (**in Chinese with English abstract**).

[4] Clark AG (2014). Genetics: The vital Y chromosome. Nature.

[5] Cortez D, Marin R, Toledo-Flores D, Froidevaux L, Liechti A, Waters PD, Grützner F, Kaessmann H (2014). Origins and functional evolution of Y chromosomes across mammals. Nature.

[6] Edwards CJ, Gaillard C, Bradley DG, MacHugh DE (2000). Y-specific microsatellite polymorphisms in a range of bovid species. Anim. Genet..

[7] Li R, Wang SQ, Xu SY, Huang JP, Wang FQ, Ma ZJ, Dang RH, Lan XY, Chen H, Lei CZ (2014). Novel Y-chromosome polymorphisms in Chinese domestic yak. Anim. Genet..

[8] Zhang, Q. In *Y chromosome molecular genetic diversity analysis in water Buffalo, yak and horse. [Master's thesis]*. China: Northwest Agriculture & Forestry University (2015).

[9] Ma ZJ, Xia XT, Chen SM, Zhao XC, Zeng LL, Xie YL, Chao SY, Xu JT, Sun YG, Li RZ, Guanque ZX, Han JL, Lei CZ (2018). Identification and diversity of Y-chromosome haplotypes in Qinghai yak populations. Anim. Genet..

[10] Ma, Z. J. In *Study on the paternal genetic diversity and origin of the yak (Bos grunniens) [doctoral dissertation]*. China: Northwest Agriculture & Forestry University (2019).

[11] Li GZ, Luo J, Chen SM, Hanif Q, He DC, Ma ZJ (2021). Maternal genetic diversity, differentiation and phylogeny of three white yak breeds/populations in China. Anim. Biotechnol..

[12] Ma ZJ, Xia XT, Chen SM, Bai M, Lei CZ, Han JL (2020). A global analysis of Y-STR *INRA189* polymorphism in chinese domestic yak breeds/populations. Anim. (Basel).

[13] Seyed Majidi A, Bazzazi H, Zamani S, Ghaemi EA (2020). Comparison of hspX gene sequence in the Beijing and non-Beijing Mycobacterium tuberculosis. J. Clin. Tuberculosis Other Mycobacterial Dis..

[14] Hall TA (1999). BIOEDIT: A user-friendly biological sequence alignment editor and analysis program for Windows 95/98/NT. Nucleic Acids Symp. Ser..

[15] Holland MM, Parson W (2011). GeneMarker® HID: A reliable software tool for the analysis of forensic STR data. J. Forens. Sci..

[16] Librado P, Rozas J (2009). DNASP v5: A software for comprehensive analysis of DNA polymorphism data. Bioinformatics.

[17] Excoffier L, Laval G, Schneider S (2007). Arlequin (version 3.0): An integrated software package for population genetics data analysis. Evol. Bioinform. Online.

[18] Kumar S, Stecher G, Tamura K (2016). MEGA7: Molecular evolutionary genetics analysis version 7.0 for bigger datasets. Mol. Biol. Evol..

[19] Bandelt HJ, Forster P, Röhl A (1999). Median-joining networks for inferring intraspecific phylogenies. Mol. Biol. Evol..

[20] Wright S (1978). Evolution and the Genetics of Populations vol. 4: Variability Within and Among Natural Populations.

