# Supplementary material for: Paternal genetic diversity, differentiation and phylogeny of three white yak breeds/populations in China
Source: Sci Rep. 2022 Nov 11;12:19331. doi: 10.1038/s41598-022-23453-w (PMC9652388; doi:10.1038/s41598-022-23453-w)
Supplement: Supplementary file 1 — Supplementary Figure S1. [file 41598_2022_23453_MOESM1_ESM.docx]

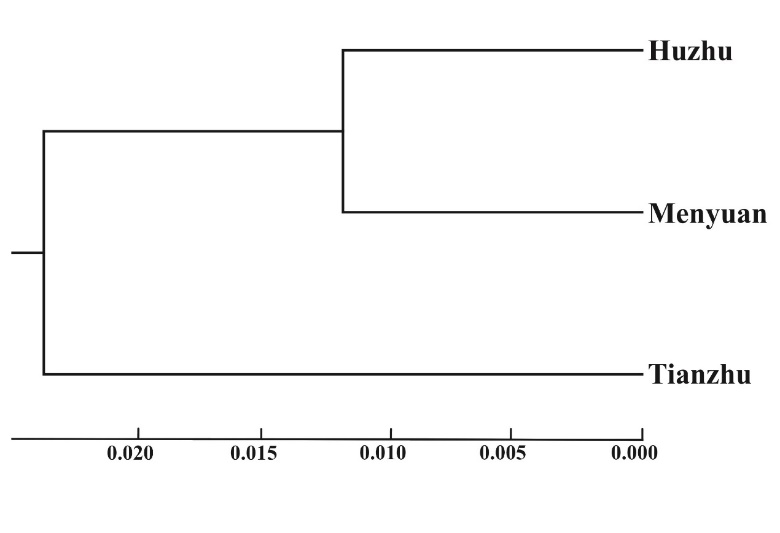


Figure S1. Clustering relationship among three Chinese white yak breeds/populations based on UPGMA method.
