# Supplementary material for: Paternal genetic diversity, differentiation and phylogeny of three white yak breeds/populations in China
Source: Sci Rep. 2022 Nov 11;12:19331. doi: 10.1038/s41598-022-23453-w (PMC9652388; doi:10.1038/s41598-022-23453-w)
Supplement: Supplementary file 2 — Supplementary Table S1. [file 41598_2022_23453_MOESM2_ESM.docx]

Table S1 Y chromosome haplotypes/haplogroups identified in three white yak breeds/populations.

| Y-SNPs | | | | | | | | | | Y-STR | Haplotype^a^ | haplogroup |
| --- | --- | --- | --- | --- | --- | --- | --- | --- | --- | --- | --- | --- |
| *SRY4* | *USP9Y* | *UTY19* | | *AMELY3* | *OFD1Y10* | | | | | *INRA189* |  |  |
| g.120 | g.249 | g.120 | g.131 | g.237 | g.306 | g.569 | g.578 | g.608 | g.653 |  |  |  |
| G＞A | C＞T | G＞A | T＞G | A＞G | C＞T | A＞C | A＞C | G＞T | G＞C |  |  |  |
| G | C | G | T | A | C | A | A | G | G | 157 | H1Y1 | Y1 |
| G | C | G | G | A | C | A | A | G | G | 157 | H9Y1 |  |
| G | C | G | G | A | C | A | A | T | G | 157 | H10Y1 |  |
| A | T | A | T | G | T | A | A | G | G | 155 | H11Y2 | Y2 |
| A | T | A | T | G | T | A | A | G | G | 157 | H12Y2 |  |
| A | T | A | T | G | T | A | A | G | G | 161 | H13Y2 |  |

*Note*: a: Y chromosome haplotypes identified by referring to the previous report from Ma et al (2019).
