# Supplementary material for: Paternal genetic diversity, differentiation and phylogeny of three white yak breeds/populations in China
Source: Sci Rep. 2022 Nov 11;12:19331. doi: 10.1038/s41598-022-23453-w (PMC9652388; doi:10.1038/s41598-022-23453-w)
Supplement: Supplementary file 3 — Supplementary Table S2. [file 41598_2022_23453_MOESM3_ESM.docx]

Table S2 *F_ST_* (Below diagonal) and *R_ST_* (Above diagonal) values among white yak breeds/populations.

| Breeds/populations | Tianzhu | Huzhu | Menyuan |
| --- | --- | --- | --- |
| Tianzhu | — | 0.0189 | 0.0826^∗^ |
| Huzhu | 0.0186 | — | 0.0000 |
| Menyuan | 0.0763^∗^ | -0.0050 | — |

*Note*: the asterisk shows P<0.05.
